# The Association Between Inflammaging and Age-Related Changes in the Ruminal and Fecal Microbiota Among Lactating Holstein Cows

**DOI:** 10.3389/fmicb.2019.01803

**Published:** 2019-08-09

**Authors:** Guoxing Zhang, Yachun Wang, Hanpeng Luo, Wenqing Qiu, Hailiang Zhang, Lirong Hu, Yajing Wang, Ganghui Dong, Gang Guo

**Affiliations:** ^1^Key Laboratory of Animal Genetics, Breeding and Reproduction, Ministry of Agriculture and Rural Affairs, National Engineering Laboratory for Animal Breeding, College of Animal Science and Technology, China Agricultural University, Beijing, China; ^2^Shenzhen Weishengtai Technology Co., Ltd., Shenzhen, China; ^3^College of Life Sciences and Bioengineering, Beijing Jiaotong University, Beijing, China; ^4^State Key Laboratory of Animal Nutrition, College of Animal Science and Technology, China Agricultural University, Beijing, China; ^5^Beijing Sunlon Livestock Development Co., Ltd., Beijing, China

**Keywords:** inflammatory cytokines, gut microbiota, inflammaging, 16S rRNA, metagenome

## Abstract

Inflammaging is well understood in the study of humans; however, it is rarely reported for dairy cows. To understand the changing pattern of the gut microbiota, inflammatory status and milk production performance during the aging process in cows, we grouped 180 cows according to their lactation period: L1 (*n* = 60, 1st lactation), L3 (*n* = 60, 3rd lactation), and L5+ (*n* = 60, at least 5th lactation) and analyzed their milk components and daily milk yields to evaluate the changing pattern of milk production. The microbiota was analyzed using high-throughput sequencing of amplicons of 16S rRNA, which also allowed us to predict the functions of microbes and then study the changing pattern of the ruminal and fecal microbiota. Serum cytokines, including TNF-α, IL-6, IL-10, and TGF-β were measured to study the progress of inflammaging in the cows. We found that old cows (L5+) suffered from a long-term and low-level chronic inflammation, as indicated by significantly higher levels of inflammatory cytokines IL-10, TNF-α, and TGF-β in the L5+ group (*p* < 0.001). We also observed a significant decrease in daily milk yield and milk lactose, as well as a significant increase in somatic cell score, among the cows in the L5+ group. For the gut microbiota, most of the genera belonging to *Prevotellaceae* and *Lachnospiraceae*, which had a higher abundance among cows of both the L1 and L3 groups (LEfSe, LDA > 2), showed a similar change pattern during the aging process, both in the rumen and in feces, and across the six farms. Beneficial bacteria, like *Bacteroidaceae*, *Eubacterium*, and *Bifidobacterium*, displayed lower abundance in the feces of the L5+ group (LEfSe, LDA > 2). Reconstruction of the fecal bacteria community indicated transformation of the fermenting pattern of older cows’ (L5+) feces microbiota, with increased functions related the protein metabolism and fewer functions related to carbohydrate and lipid metabolism compared with those in L1 (*p* < 0.05). Finally, the connections among these changing patterns were revealed using redundancy analysis and network analysis. The results support the hypothesis of prolonging a cows’ productive life and improve dairy cow milk productive performances by manipulating the gut microbiota.

## Introduction

Over the past few decades, scientists have emphasized the importance of the ruminal microbiome in ruminant digestion. Ruminants depend on ruminal microbes to decompose feed into micromolecules [such as volatile fatty acids (VFAs) and ammonia], which are easily absorbed by the host. Few studies have investigated the fecal bacteria communities, because fecal bacteria communities have fewer effects on cows digestive ability compared with that of the ruminal bacteria communities. Until recently, the gut microbiota (refers mainly to the ruminal and fecal microbiota in our discussions about cow microbiota) was considered as much more than a community to help with digesting foods. Some studies found that the ruminal microbial ecosystem plays an important role in the development of diseases, such as subacute ruminal acidosis (Wetzels et al., 2017) and frothy bloat (Pitta et al., 2016). Other studies reported the correlation between fecal microbiota and mastitis (Ma et al., 2016) and milk composition (Zhang et al., 2017). Thus the relationship between the gut microbiota and host health cannot be ignored; however, the relationship between the composition of the fecal microbiota and host inflammaging, which has been frequently reported in other species, remains unknown for dairy cows.

The gut microbiome is associated with many of the most discussed topics in human health, such as aging (OToole and Jeffery, 2015), cancer (Roy and Trinchieri, 2017), metabolic diseases (obesity, diabetes) (Sonnenburg and Bäckhed, 2016), the digestive system (Desai et al., 2016), the cardiovascular system (Zhu et al., 2016), the immune system (Thaiss et al., 2016), and the central nervous system (Mayer et al., 2014). These studies also confirmed the symbiotic relationship between host and its gastrointestinal microbes. On the one hand, the gut microbiome produces a wide array of microbial metabolites [e.g., short-chain fatty acids (SCFA) and trimethylamine-N-oxide (TMAO)], which might permeate into the circulatory system, where they might have either beneficial (Malmuthuge and Guan, 2016) or toxic (Zhu et al., 2016) effects on host health. On the other hand, hosts are able to limit the activities of microbes and shape the structure of the microbiome through the host immune system (Thaiss et al., 2016), gene expression, or the manipulation of the diet using direct-fed microbials, prebiotics, or probiotics (Malmuthuge and Guan, 2017).

Older populations suffer from a chronic low-grade inflammation, known as inflammaging, which is characterized by a higher level of inflammation-related cytokines compared with those in younger people, and this inflammaging might be the cause of debility and diseases of old age (Biagi et al., 2010; Claudio, 2010). There are many factors that can result in inflammaging, and these factors may be involved with the genetic effects of mitochondrial DNA variants, changes in eating habits, and chronic exposure to antigens (Claudio, 2010). The phylogenetic composition of the bacterial community changes along with the process of aging. The latest studies suggested that inflammaging could also be resulted from age-related microbiota changes. Floris showed by transferring aged microbiota to young germ-free (GF) mice that certain bacterial species (e.g., *Akkermansia*, *Proteobacteria*) within the aged microbiota promote inflammaging (Floris et al., 2017). Thevaranjan demonstrated that inflammaging was highly associated with age-related microbiota changes that might drive intestinal permeability and decrease macrophage function (Thevaranjan et al., 2017). Inflammaging can undermine the balance between gut microbiota and gut-associated immune system (Biagi et al., 2010), and contribute to the development of a number of age-related chronic diseases such as atherosclerosis, type 2 diabetes, Alzheimer’s disease, osteoporosis, and major depression (Claudio, 2010). The remodeling of gut microbiota may help with alleviating inflammaging and attaining longevity. The life span of middle-age killifish, whose gut was recolonized with bacteria from young donors, was extended (Smith et al., 2017). However, the theory about which (and how) bacteria influence inflammaging is not fully clear yet. A review (Brüssow, 2013) summarized three groups of gut bacteria according to their effects on host health: (1) Symbiotic gut bacteria, such as *Bacteroidaceae*, *Eubacterium*, *Peptococcaceae*, *Bifidobacterium*, and *Lactobacillus*, are beneficial to human health; (2) a second group, comprising *Escherichia*, *Streptococcus*, and *Veillonella*, produce toxic compounds by fermenting proteins, possibly leading to aging; and (3) pathogenic gut bacteria that induce infections.

Older dairy cows are more likely to face various health problems, as well as reduced production efficiency, resulting in a higher culling hazard (Maia et al., 2014) and causing a measurable economic loss. The natural lifespan of a cow can be up to 25 years; however, the average culling age for dairy cows worldwide is only 6 years old mostly due to the physiological and psychological stress of lactating cows. Despite the importance of longevity to the productivity of cows, as well as to animal welfare, there is a lack of proof supporting the hypothesis that a cows’ productive life could be prolonged by manipulating the gut microbiota. Some studies reported that the ruminal microbiota changed during newborn maturation or with increasing age (Jami et al., 2013; Liu et al., 2017). Studies also confirmed the relationship between the rectal microbiota and the maturation of newborn dairy calves (Mayer et al., 2012; Alipour et al., 2018). However, no study has connected variations in the fecal microbiota with inflammaging, nor determined whether this could be applied to different farm environments.

The development of high-throughput sequencing has allowed us to gain a full-scale view of the composition (by sequencing 16S rRNA amplicons) and function (by the sequencing the metagenome or by prediction from 16S rRNA data) of the gut community. However, how to handle the sequences derived from sequencing of 16S rRNA amplicons remains a challenge. The operational taxonomic unit (OTU), often at a similarity of 0.97, was developed to define clusters of organisms (which could be uncultivated or unknown) grouped by their DNA (Blaxter et al., 2005). The analysis of 16S rRNA amplicons based on OTUs has dominated the field of microbiota study for more than 10 years. There is, however, a need to identify higher phylogenetic resolution OTUs and to combine OTU results from different studies; therefore, denoising methods, such as Deblur (Amir et al., 2017), have been developed to obtain putative error-free sequences and identify sub-operational-taxonomic-units (sOTUs).

In the present study, we used advanced technique to explore the variations of the fecal bacteria communities, milk composition, inflammatory cytokines, and routine blood analysis of 180 cows from six dairy farms. We also analyzed the rumen bacterial communities of 30 cows, with the aim of finding an explanation for the fragility of older dairy cows, and the relationship between the cow gut microbiota and inflammaging, as well as longevity.

## Materials and Methods

### Ethics Statement

The Institutional Animal Care and Use Committees (IACUCs) approved all the experimental procedures, which complied with the China Physiological Society’s guiding principles for research involving animals. This study did not involve any endangered or protected animal species, and did not cause any harm to the experimental animals.

### Experimental Design and Sample Collection

Healthy female Holstein cattle (*n* = 180) of different ages were obtained from six different farms (F1–F6, 30 cows from each farm). The cows were selected according to their physiological status and grouped by lactation period as follows: L1 (*n* = 60, 1st lactation), L3 (*n* = 60, 3rd lactation), and L5+ [*n* = 60, ranging from the 5th lactation to the 9th lactation (average 6th lactation)]. Each farm provided 10 cows of group L1, 10 cows of group L3 and 10 cows of group L5+. The cows from same farm had been fed with exactly the same total mixed ration diet for over 1 month, and the cows from different farms were fed with different total mixed ration diets; however, all diets were based on corn silage, and all the cows were fed *ad libitum*. All six dairy farms are located in Beijing, China, and belong to Beijing Sunlon Livestock Development Ltd., and thus had a similar breeding mode. There were no significant differences among the three groups in terms of rectal temperature (the mean of two independent measurements in the morning), days in milk (DIM), and body condition score (the mean of two independent evaluations) (Table 1). None of the cows were diagnosed with any diseases and had not been treated with any antibiotics for the last 3 months until sample collection.

**TABLE 1:** The body condition score, rectal temperature, and days in milk of cows in this study.

**Item**	**Least squares mean ± SE**			***P*-value**
		
	**L1 (*n* = 60)**	**L3 (*n* = 60)**	**L5+ (*n* = 60)**	
Body condition score	2.9 ± 0.09	2.8 ± 0.09	2.9 ± 0.09	0.668
Rectal temperature (°C)	38.8 ± 0.05	38.8 ± 0.05	38.8 ± 0.05	0.548
Days in milk (days)	216.6 ± 8.53	213.4 ± 8.53	225.6 ± 8.53	0.576

The daily milk yield (DMY) was recorded three times in three consecutive months before sample collection, and the mean was calculated for further comparison. Sample collection began in March 2017 and ended in August 2017, with the samples (feces, rumen liquid, milk, and blood) coming from the same farm being collected within 24 h. Feces were collected aseptically in the morning and transported to the laboratory immediately on dry ice before being stored in the refrigerator at −80°C. Milk samples were collected in the morning, afternoon, and evening, followed by mixing in the proportion of 4:3:3. Preservatives were used to keep the milk samples fresh. In addition, 10 ml of anticoagulant and 10 ml non-anticoagulant blood samples were collected at the tailhead of cows. Non-anticoagulant blood samples were coagulated at room temperature for 1 h before centrifugation at 1,000 × *g* for 10 min to separate the serum from the erythrocytes, and the sera were stored at −20°C until further processing.

The rumen liquid of 30 cows could only be collected from the first farm (F1) because of the lack of conditions to collect the ruminal liquid at the other farms; however, we successfully collected feces, milk, and blood samples of all 180 cows selected in the current study. The rumen liquid was collected at noon (approximately 4 h after the morning feed and on the same day as feces were collected from the cows in F1) using a flexible stomach tube, which was washed with clean water before each collection. About 50 mL of rumen liquid from each cow was aspired through the mouth, with the initial 100 mL (approximately) discarded to avoid contamination by saliva. The obtained sample was transported to the laboratory on ice and then stored in at −80°C immediately. The whole period of rumen liquid collection and storage lasted less than 3 h.

### The Measurement of Cytokines and Milk Components, and Routine Blood Analysis

Inflammation-related cytokines in serum including tumor necrosis factor alpha (TNF-α), interleukin (IL)-6, transforming growth factor beta (TGF-β), and IL-10, were determined using an ST-360 Microplate Reader (Kehua Bio-engineering Co., Ltd., Shanghai, China) and cytokine diagnostic reagents (Jinhaikeyu Biological Technology Development Co., Ltd., Beijing, China), which is based on an enzyme-linked immunosorbent assay (ELISA). Routing blood analysis, including WBC (white blood cell count), W-SCR (lymphocyte cell ratio), W-MCR (mononuclear cell rate), W-LCR (granulocyte cell ratio), W-SCC (lymphocyte cell count), W-MCC (mononuclear cell count), W-LCC (granulocyte cell count), RBC (red blood cell count), HGB (hemoglobin), HCT (hematocrit), MCV (mean corpuscular volume), MCH (mean corpuscular hemoglobin), MCHC (mean corpuscular hemoglobin concentration), RDW-CV (red blood cell distribution width of coefficient of variation), RDW-CV (red blood cell distribution width of standard deviation), PLT (platelet count), PDW (platelet distribution width), MPV (mean platelet volume), and P-LCR (large cell ratio of platelet) in anticoagulant whole blood were analyzed using a K-4500 Fully Automated Hematology Analyzer (Sysmex Corporation, Kobe, Japan) within 24 h after sample collection. The component analysis models designed by Bentley Instruments Inc. (Chaska, MN, United States) were used to measure and calculate milk components, including Lactose (milk lactose), Car (milk carbamide), FPD (freezing point depression of milk), Solids (milk solids), Fat (milk fat), Protein (milk protein), SCS (somatic cell score of milk), and this procedure was completed within 48 h after milk sample collection. All the steps referred to above were operated according to corresponding manufacturer’s instructions.

### Determination of Ruminal and Fecal Bacteria Communities

The procedures of DNA extraction, amplification, and sequencing were completed by Novogene Bioinformatics Technology Co., Ltd. (Beijing, China). DNA was extracted from 0.2 g of feces (5 mL for the rumen liquid) using a QIAamp DNA Stool Mini Kit (Qiagen, Hilden, Germany). Rumen liquid was first centrifuged at 8,000 × g for 20 min, and the supernatant was removed. Feces (pellet after centrifugation for rumen liquid) was added into 1 ml of lysis buffer (500 mM NaCl, 50 mM Tris−HCl pH 8, 50 mM EDTA, 4% SDS) that blended with mini Bead Beater (BioSpec, Bartlesville, OK, United States), and treated in FastPrep (MP Biomedicals, Irvine, CA, United States) at 5,000 oscillations per minute for 60 s. The mixture was heated at 95°C for 15 min, then centrifuged for 5 min at 12,000 × *g* to pellet particles. 250 ml of 10 M ammonium acetate were added to the supernatant, followed by incubation in ice for 5 min and centrifugation at 12,000 × *g* for 10 min. One volume of isopropanol was added to each sample and incubated in ice for 30 min. Precipitated nucleic acids were collected by centrifugation for 15 min at 12,000 × *g* and washed with ethanol 70%. Pellet was suspended in 100 ml of TE buffer and treated with 2 ml of DNase-free RNase (10 mg/ml) at 37°C for 15 min. Protein removal by Proteinase K treatment and DNA purification with QIAamp Mini Spin columns were performed following the kit protocol. The final DNA concentration was determined using a NanoDrop 2000 UV-vis spectrophotometer (Thermo Fisher Scientific, Wilmington, NC, United States), and DNA quality was checked using 1% agarose gel electrophoresis. The V3–V4 (341F 5′-CCTAYGGGRBGCASCAG-3′, 806R 5′-GGACTACNNGGGTATCTAAT-3′) region of the 16S ribosomal RNA (rRNA) gene was amplified using a thermocycler PCR system (GeneAmp 9700, ABI, Foster City, CA, United States). The PCR reactions were conducted using the following program: 3 min of denaturation at 95°C; 27 cycles of 30 s at 95°C, 30 s for annealing at 55°C, and 45 s for elongation at 72°C; and a final extension at 72°C for 10 min. The PCR reactions were performed in triplicate in a 20 μL mixture containing 4 μL of 5 × FastPfu Buffer, 2 μL of 2.5 mM dNTPs, 0.8 μL of each primer (5 μM), 0.4 μL of FastPfu Polymerase, and 10 ng of template DNA. The resulting PCR products were extracted from a 2% agarose gel and further purified using the AxyPrep DNA Gel Extraction Kit (Axygen Biosciences, Union City, CA, United States) and quantified using QuantiFluor-ST (Promega, Madison, WI, United States) according to the manufacturer’s protocol. Purified amplicons were pooled in equimolar amounts and paired-end sequenced (2 × 250) on an Illumina (San Diego, CA, United States) HiSeq 2500 instrument (*n* = 61,645 ± 9,513 (mean ± SD) raw reads per fecal sample, *n* = 48,815 ± 6,590 per ruminal liquid sample) according to the standard protocols. The Q30 of the raw reads was 96 ± 0.86 (mean ± SD). All the bioinformatic analysis procedures of the sequences were completed on a QIIME 2 data science platform (Bolyen et al., 2018). Paired-end sequences were merged and quality-filtered using the approaches recommended by QIIME 2; reads with an average Phred quality score below 20 were filtered out, and paired-end reads were merged at a minimum overlap of 15 nt. The sub-operational-taxonomic-unit (sOTU) is an OTU at a similarity of 100%, and thus two sequences from the same region of 16S rRNA with a single-nucleotide difference belong to two different sOTUs. Deblur, constructed recently by Amir et al. (2017), and integrated to QIIME 2, was used to obtain putative error-free sequences and pick sOTUs. After the default procedure of Deblur, we obtained a total of 4,115,142 reads for the fecal samples [22,862 ± 6,588 (mean ± SD) per sample], and 398,597 reads for the ruminal samples (13,287 ± 1,870), with no chimeras and no sequencing errors (theoretically). After filtering the sOTUs observed in less than 10 samples, 4,745 sOTUs (3,301 sOTUs for rumen liquid) with single-nucleotide differences and hardly any false positives were obtained. The representative sequence of each sOTU was then aligned to the Silva database (Christian et al., 2013) (Release 132) (trimmed to the V3–V4 region bound by the 341F/806R primer pair) to assign taxonomy using the q2-feature-classifier (Bokulich et al., 2018). Any contaminating mitochondrial and chloroplast sequences were filtered out using the QIIME2 feature-table plugin. This resulted in 86% of the total sequences being assigned taxonomically at the genus level. The closed-reference OTUs were then chosen and further used to predict the functional profile of bacterial communities and to identify Kyoto Encyclopedia of Genes and genomes (KEGG) pathways using PICRUSt (Langille et al., 2013). Unless specified above, the parameters used in the analysis were set to the defaults.

### Statistical Analysis

To study how the production performance, inflammatory cytokines, and blood biochemical indexes changed with increasing of age, an SAS (Littell et al., 1996) general linear model (GLM) procedure considering the fixed effects of farm, lactation stage (two stages decided by the median of days in milk), lactation period (*L1, 1st lactation; L3, 3rd lactation; L5*+*, at least 5th lactation*), and the interaction between farm and lactation period, was used. Principal coordinates analysis (PCoA) of Bray–Curtis dissimilarity (Bray and Curtis, 1957), which was calculated from sOTU sequence count table, was used to visualize the difference in the bacterial community between samples and among groups, followed by Permutational Multivariate Analysis of Variance (PERMANOVA), a non-parametric multivariate statistical test (Anderson, 2010), to test the significance of the difference in bacterial community among lactation groups.

Before the following analysis of bacteria and predicted pathways, a relative abundance table of bacteria at genus level or KEGG pathways of the third level were calculated, and the mean-only version of a non-parametric empirical Bayes method (Johnson et al., 2007; Leek et al., 2012) was used to remove the effect of uninterested variables (farm groups). The non-strict version of Linear discriminant analysis Effect Size (LEfSe), which determines significant taxa differing in at least one (and possibly multiple) class value(s) (Segata et al., 2011), was then used to identify the bacterial biomarkers of lactation groups (*L1, 1st lactation; L3, 3rd lactation; L5*+*, at least 5th lactation*). The predicted KEGG pathways observed in less than 90 samples were filtered out, as the relative abundance of these pathways deviated markedly from the normal distribution. ANOVA, followed by Duncan’s test, was then used to detect the pathways that were differently abundant across the three lactation groups after the relative abundance of pathways was log transformed and scaled to a standard normal distribution. Redundancy Analysis (RDA), introduced by Borcard (Borcard et al., 2011), was used to analyze the correlation between the cows’ physiological indexes (inflammatory cytokines, milk production performance, routine bloods) measured in this study, and bacterial communities. The permutation test method (function envfit) constructed in the R VEGAN package (Dixon, 2003) was then used to test the significance of this correlation. Spearman’s rank correlation coefficient was calculated to measure the relationship among the cows’ physiological indexes, ruminal bacteria, and fecal bacteria.

### Nucleotide Sequence Accession Numbers

The sequences in this study were accessible from the NCBI Sequence Read Archive with accession number SRP202074.

## Results

### Changes of Inflammation-Related Cytokines, Routine Bloods, and Production Performances With Increasing Age

Four cytokines (TNF-α, IL- 6, TGF-β, and IL-10) were measured to study the changes in inflammation-related cytokines with increasing age, and to determine if aged cows suffer from inflammation. Using SAS GLM, we observed low-level inflammation among the cows in the L5+ group. The concentrations of TNF-α, TGF-β, and IL-10 showed a similar changing pattern; their levels were higher in L5 than L1 and L3 (*p* < 0.001) but not statistically different between L1 and L3 (Table 2). IL-6 levels were significantly higher in the L3 and L5+ groups, and no significant difference was observed between L3 and L5+. Thus, the levels of all the measured cytokines were significantly higher in L5+ compared with those in L1 (*p* < 0.05).

**TABLE 2:** Difference in inflammation-related cytokines, production performance, and routine bloods across three lactation groups.

**Item**	**Least squares mean ± SE**			***P*-value**
		
	**L1 (*n* = 60)**	**L3 (*n* = 60)**	**L5+ (*n* = 60)**	
**Inflammation-related cytokines**				
TNF-α (pg/ml)	169.29 ± 3.61^b^	166.29 ± 3.62^b^	188.44 ± 3.60^a^	<0.001
IL-6 (pg/ml)	138.36 ± 4.12^b^	154.48 ± 4.13^a^	150.55 ± 4.12^a^	0.018
IL-10 (pg/ml)	20.74 ± 0.77^b^	22.34 ± 0.77^b^	26.44 ± 0.77^a^	<0.001
TGF-β(ng/ml)	66.30 ± 1.87^b^	70.68 ± 1.87^b^	76.55 ± 1.86^a^	<0.001
**Performances of production**				
DMY (kg/day)	37.91 ± 0.83^b^	39.95 ± 0.99^a^	35.23 ± 0.84^c^	<0.001
Lactose (%)	5.00 ± 0.04^a^	4.79 ± 0.04^b^	4.67 ± 0.04^c^	<0.001
Car (mg/dL)	12.67 ± 0.44	12.66 ± 0.44	12.57 ± 0.44	0.986
FPD (m^O^H)	554.16 ± 1.65^b^	556.07 ± 1.66^b^	564.48 ± 1.64^a^	<0.001
Solids (%)	11.18 ± 0.15	11.55 ± 0.15	11.13 ± 0.15	0.090
Fat (%)	3.00 ± 0.09	3.26 ± 0.09	3.17 ± 0.09	0.146
Protein (%)	3.14 ± 0.02	3.19 ± 0.02	3.11 ± 0.02	0.065
SCS	2.95 ± 0.15^c^	3.50 ± 0.15^b^	4.49 ± 0.15^a^	<0.001
**Blood routine**				
WBC (10^9^/L)	11.67 ± 0.44	11.10 ± 0.44	10.58 ± 0.44	0.221
RBC (10^12^/L)	6.03 ± 0.07^a^	5.73 ± 0.07^b^	5.70 ± 0.07^b^	<0.001
HGB (g/L)	96.59 ± 1.15	93.78 ± 1.15	93.88 ± 1.15	0.151
HCT (%)	27.90 ± 0.33	27.03 ± 0.33	26.80 ± 0.33	0.051
MCV (fL)	47.67 ± 0.31^b^	48.75 ± 0.31^a^	48.65 ± 0.31^a^	0.024
MCH (pg)	16.52 ± 0.12^b^	16.96 ± 0.12^a^	17.04 ± 0.12^a^	0.004
MCHC (g/L)	346.93 ± 0.87	347.79 ± 0.87	349.77 ± 0.87	0.064
PLT (10^9^/L)	429.27 ± 13.38	418.09 ± 13.40	390.67 ± 13.35	0.113
W-SCR (%)	56.23 ± 1.00	53.60 ± 1.00	53.54 ± 1.00	0.100
W-MCR (%)	6.00 ± 0.21	6.18 ± 0.21	6.51 ± 0.21	0.210
W-LCR (%)	37.77 ± 0.96	40.22 ± 0.96	39.94 ± 0.95	0.146
W-SCC (10^9^/L)	6.69 ± 0.29	6.09 ± 0.29	5.87 ± 0.29	0.124
W-MCC (10^9^/L)	0.70 ± 0.03	0.67 ± 0.03	0.68 ± 0.03	0.800
W-LCC (10^9^/L)	4.28 ± 0.17	4.34 ± 0.17	4.03 ± 0.17	0.384
PDW (fL)	6.64 ± 0.09^b^	6.78 ± 0.09^ab^	6.98 ± 0.09^a^	0.020
MPV (fL)	6.12 ± 0.05^b^	6.26 ± 0.05^ab^	6.33 ± 0.05^a^	0.006
RDW-SD	17.46 ± 0.29	17.70 ± 0.29	18.29 ± 0.29	0.123
RDW-CV	0.17 ± 0.00	0.17 ± 0.00	0.17 ± 0.00	0.265
P-LCR (%)	3.21 ± 0.20^b^	3.88 ± 0.20^a^	3.89 ± 0.20^a^	0.024

*TNF-α, tumor necrosis factor alpha; IL-6, interleukin 6; TGF-β, transforming growth factor beta; IL-10, interleukin 10; DMY, daily milk yield; Lactose, milk lactose; Car, milk carbamide; FPD, freezing point depression of milk; Solids, milk solids; Fat, milk fat; Protein, milk protein; SCS, somatic cell score of milk; WBC, white blood cell count; W-SCR, lymphocyte cell ratio; W-MCR, mononuclear cell rate; W-LCR, granulocyte cell ratio; W-SCC, lymphocyte cell count; W-MCC, mononuclear cell count; W-LCC, granulocyte cell count; RBC, red blood cell count; HGB, hemoglobin; HCT, hematocrit; MCV, mean corpuscular volume; MCH, mean corpuscular hemoglobin; MCHC, mean corpuscular hemoglobin concentration; RDW-CV, red blood cell distribution width of coefficient of variation; RDW-CV, red blood cell distribution width of standard deviation; PLT, platelet count; PDW, platelet distribution width; MPV, mean platelet volume; and P-LCR, large cell ratio of platelet were compared across three lactation groups (L1, L3, L5+, corresponding to 1st lactation, 3rd lactation, and at least 5th lactation) using GLM, general linear model in SAS. The least squares means labeled with different letters were significantly different (Duncan’s test, *p* < 0.05), otherwise they were not significantly different.*

SAS GLM analysis of milk production performance suggested a decrease in milking performance as the cows grew older. Milk lactose and DMY tended to decline, while the SCS and FPD tended to increase with age (*p* < 0.001). Among the three groups, L5+ had the lowest level of daily milk yield, at about 5 kg lower than that in L3, and 3 kg lower than that in L1 (*p* < 0.05). Total milk solids and milk protein levels were also the lowest in L5+ when comparing the means, although the differences were not significant. This decline in production performance of old dairy cows (L5+) indicated that the physiological functions of aged dairy cows had diminished.

We also compared the blood physiology of the three groups. As shown in Table 2, there was a significant decline in the RBC count in L3 and L5+ (*p* < 0.001). Some blood physiological indexes related to the size of red blood cells (MCV, MCH) and the size of platelets (PDW, MPV, P-LCR) were increased in the L5+ group compared with those in the L1 group (Table 2, *p* < 0.05).

**FIGURE 1:**
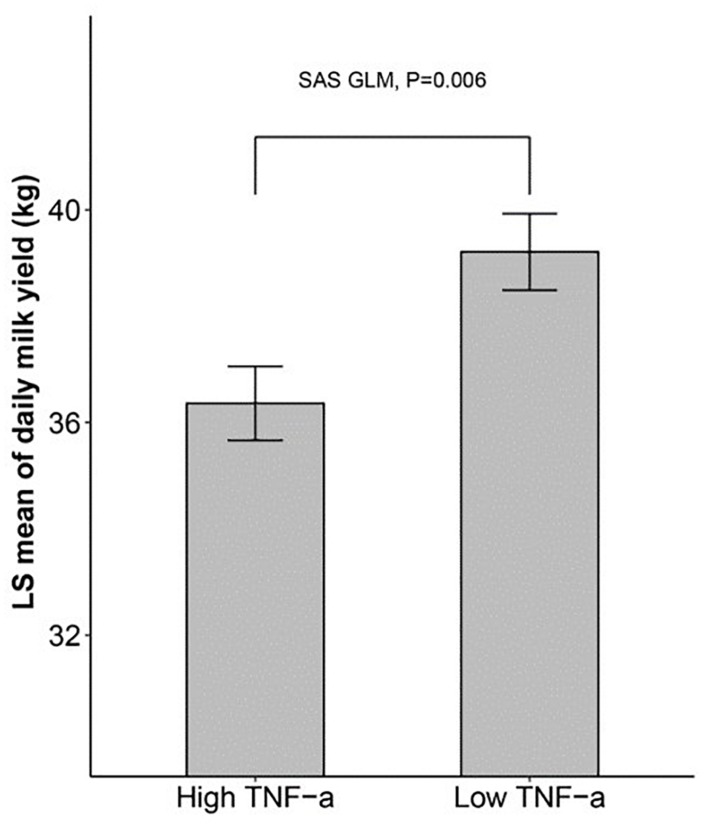
The dairy milk yield of cows with high and low TNF-α level SAS GLM procedure showing that cows with higher TNF-α levels produce less milk. 180 cows were divided into two groups at the median of TNF-α level, and the daily milk yield of the two groups was compared using GLM (General Linear Model) in SAS.

We then sought to determine if there is a connection between inflammatory cytokines and milk yield. We divided the 180 cows into two groups exactly at the median of the TNF-α level, and then compared the milk yield between the two groups using SAS GLM, which considered farm group, lactation group (L1, L3, L5+), lactation stage (two levels divided by the median of lactation days), and TNF-α group as fixed effects. The results in Figure 1 showed that the dairy cows with a higher level of blood TNF-α in blood tended to produce less milk (about 2 kg, *p* < 0.01).

### Variation of Fecal Microbiota Among the Six Farms

Bray–Curtis dissimilarity (Bray and Curtis, 1957), which is computed from sequence counts of bacteria, is good at quantifying the difference in abundance between two different bacterial communities. To make this Bray–Curtis dissimilarity easier to understand, we used the technique of unsupervised principal coordinates analysis (PCoA) to visualize the Bray–Curtis dissimilarity matrix among 180 dairy cows’ feces microbiota. In the PCoA plot (Supplementary Figure S1A), the visible distance between two points approximates to the Bray–Curtis dissimilarity between the two corresponding cows’ bacteria communities. A remarkable spatial separation among the six farms’ clouds was observed in Supplementary Figure S1A (PERMANOVA, *p* < 0.001), suggesting a large difference between the cows’ feces microbiota from different farms.

To further understand this variation in fecal microbiota between different farms, we used a flower figure (Supplementary Figure S1B) to characterize the distribution of sOTUs among the six farms. Supplementary Figure S1B shows that, although most of the sOTUs (54%) were shared by all farms among the 4745 observed sOTUs, there were some sOTUs observed only in one specific farm. Considering that all the sOTUs observed in less than 10 samples were excluded from this analysis, these farm-specific sOTUs could be very convincing and meaningful. More information about these common sOTUs and farm-specific sOTUs is shown in Supplementary Table S1. In addition, even for those sOTUs that were shared among most farms, their abundance fluctuated markedly between farms (Supplementary Figure S2).

### Change of Fecal Bacterial Communities With Increasing Age

**FIGURE 2:**
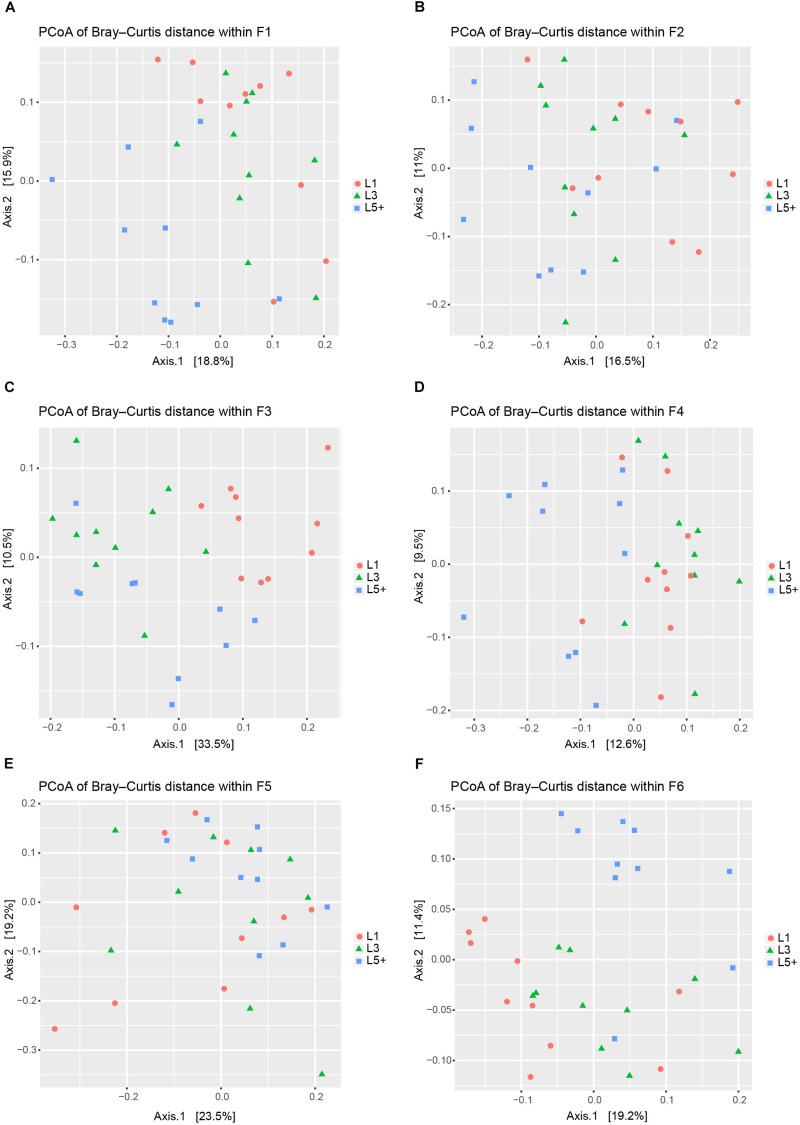
Principal coordinates analysis (PCoA) to visualize the differences among the fecal bacteria communities of three lactation groups from six farms. **(A–F)** PCoA was applied six times separately for the six farms (F1–F6) to visualize the differences in fecal bacterial communities across three lactation groups. In each plot, each point represents a sample, the distance between two points approximates the difference of their bacterial communities (Bray–Curtis dissimilarity), and the points belonging to different lactation groups were shown in different colors.

Principal coordinates analysis based on Bray–Curtis dissimilarity was applied six times independently for the six farms to analyze the difference in bacterial composition across the three lactation groups. The PCoA plots (Figure 2) showed that the clouds derived from the L1 and L5+ data were separated from each other within each farm, indicating that their bacterial communities were different. The point clouds of L3 groups, which were more likely to be located between L1 and L5+, were hard to tell from L1 in some cases (Figures 2A,D,E). We then used the PERMANOVA technique to test if there were significant separations across the centers of the three lactation groups’ bacterial communities. As shown in Table 3, five out of six farms had overall *p*-values below 0.001, which provided sufficient proof that there were significant differences across the bacterial communities from the three lactation groups. Even for the non-significant farm (F5), the difference was significant if we only looked at the *p*-value of L1 versus L5+ (*p* < 0.05). The results of PERMANOVA confirmed those of PCoA; that there was a significant difference between the bacterial composition of the L1 and L5+ groups (*p* < 0.05).

**TABLE 3:** PERMANOVA to test the difference in bacterial communities across three lactation groups.

**Farm**	**Pairwise permanova *p*-value**			**Overall *p*-value**
		
	**L1−L3**	**L1−L5+**	**L3−L5+**	
	**(*n* = 20)**	**(*n* = 20)**	**(*n* = 20)**	
F1 (*n* = 30)	0.407	<0.001	0.002	<0.001
F2 (*n* = 30)	0.005	0.007	0.035	<0.001
F3 (*n* = 30)	<0.001	<0.001	0.016	<0.001
F4 (*n* = 30)	0.017	<0.001	<0.001	<0.001
F5 (*n* = 30)	0.484	0.019	0.350	0.131
F6 (*n* = 30)	0.030	<0.001	<0.001	<0.001

*Permanova, a non-parametric method, was applied separately in six farms to test the significance of the difference in fecal bacteria communities across three lactation groups. F1–F6 represent six farm groups, and L1 (1st lactation), L3 (3rd lactation), and L5+ (at least 5th lactation) represent the three lactation groups. All the *p*-values were calculated by permutating 1000 times. Each pairwise *p*-value was calculated from 20 samples, and each overall *p*-value was calculated from 30 samples in the corresponding farm.*

**FIGURE 3:**
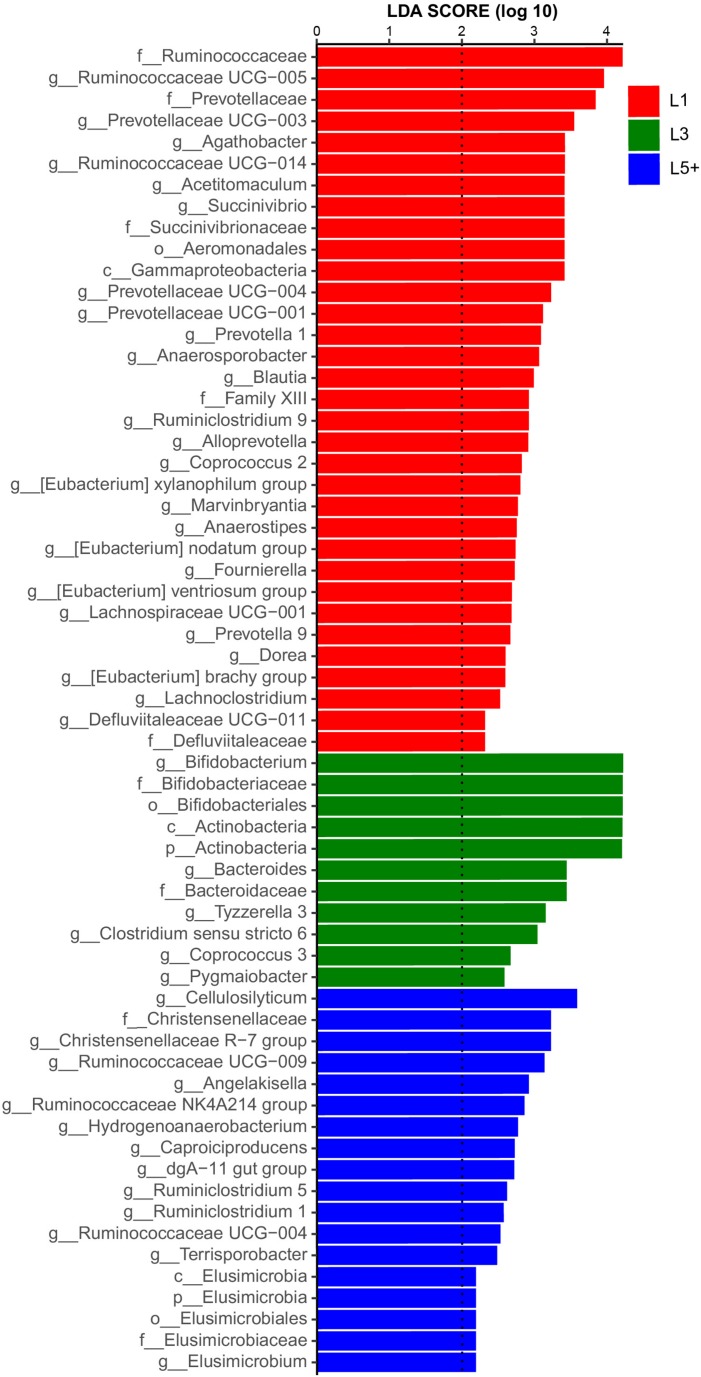
Histogram of the LDA scores computed for differentially abundant fecal bacteria across three lactation groups. LEfSe scores could be interpreted as the degree of consistent difference in relative abundance of the analyzed fecal bacteria communities across the three lactation groups. The histogram thus identifies which clades among all those detected as statistically and biologically differentially abundant and could explain the greatest differences across the three groups. In brief, the blue bars, for example, represent the bacteria with the highest abundance in L5+ compared with those in the other two groups.

**FIGURE 4:**
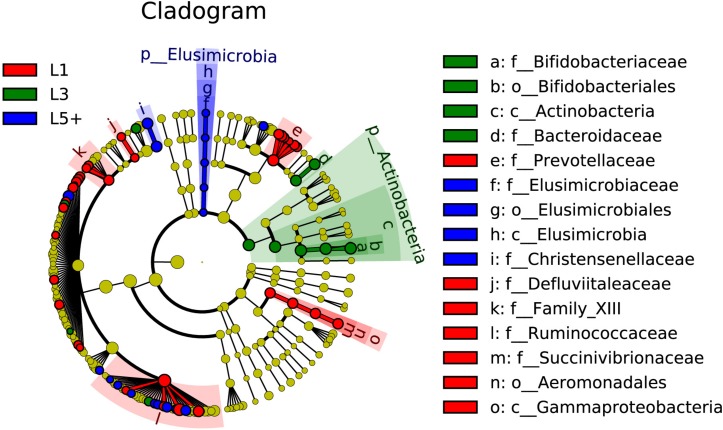
The fecal bacteria (highlighted by small circles and by shading) showing different abundance values among three lactation groups. There are six layers from the inside of this plot to the outside, corresponding to six levels of taxonomy (kingdom, phylum, class, order, family, and genus). Each node (small circle) represents a taxon; blue nodes represent the bacterial biomarkers of L5+ with the highest abundance in L5+ compared with that in the other two groups (red for 1st lactation, green for 3rd lactation), while yellow nodes indicate the bacteria that are not statistically and biologically differentially abundant among the three lactation groups. The diameter of each circle is proportional to the taxon’s abundance. This representation, employing the Silva taxonomy, simultaneously highlights high-level trends and specific genera; for example, multiple differentially abundant sibling taxa are consistent with the variation of the parent clade.

The non-strict version of LEfSe (Segata et al., 2011) was used to determine the bacteria most likely to explain the differences among lactation groups, by coupling Kruskal–Wallis tests for statistical significance with additional tests assessing biological consistency and effect relevance. Bacteria with LDA scores greater than 2 were speculated to have a different abundance across the three lactation groups (Figures 3, 4). Finally, we identified 18 clades as biomarkers of L5+, which could distinguished L5+ from the other two groups and were more abundant in the L5+ samples, including *Terrisporobacter*, *Cellulosilyticum*, *Christensenellaceae R-7 group*, *dgA-11 gut group*, and eight genera belong to the *Ruminococcaceae* (*Ruminococcaceae UCG-009*, *Ruminococcaceae UCG-004*, *Ruminococcaceae NK4A214 group*, *Ruminiclostridium 5*, *Ruminiclostridium 1*, *Hydrogenoanaerobacterium*, *Caproiciproducens*, and *Angelakisella*), as well as a lineage of *Elusimicrobia*. We found 11 clades that were more abundant in the L3 group, including *Pygmaiobacter*, *Tyzzerella 3*, *Coprococcus 3*, *Clostridium sensu stricto 6*, *Bacteroides*, and a lineage of *Bifidobacterium*. We also found 33 clades as biomarkers of L1, including *Succinivibrio*, *[Eubacterium] nodatum group*, *[Eubacterium] brachy group*, *Defluviitaleaceae UCG-011*, four genera belonging to the *Ruminococcaceae* (*Ruminococcaceae UCG-014*, *Ruminococcaceae UCG-005*, *Ruminiclostridium 9*, and *Fournierella*), 12 genera belonging to the *Lachnospiraceae* (*Marvinbryantia*, *Lachnospiraceae UCG-001*, *Lachnoclostridium*, *Dorea*, *Coprococcus 2*, *Blautia, Anaerostipes*, *Anaerosporobacter*, *Agathobacter Acetitomaculum*, *[Eubacterium] xylanophilum group*, and *[Eubacterium] ventriosum group*) and all the genera belong to the *Prevotellaceae* (*Prevotellaceae UCG-004*, *Prevotellaceae UCG-003*, *Prevotellaceae UCG-001*, *Prevotella 9*, *Prevotella 1*, and *Alloprevotella*). Clearly, there was a reconstruction within the family *Ruminococcaceae* as the cows grew older.

### Changing Pattern of Bacterial Functions With Increasing Age

**FIGURE 5:**
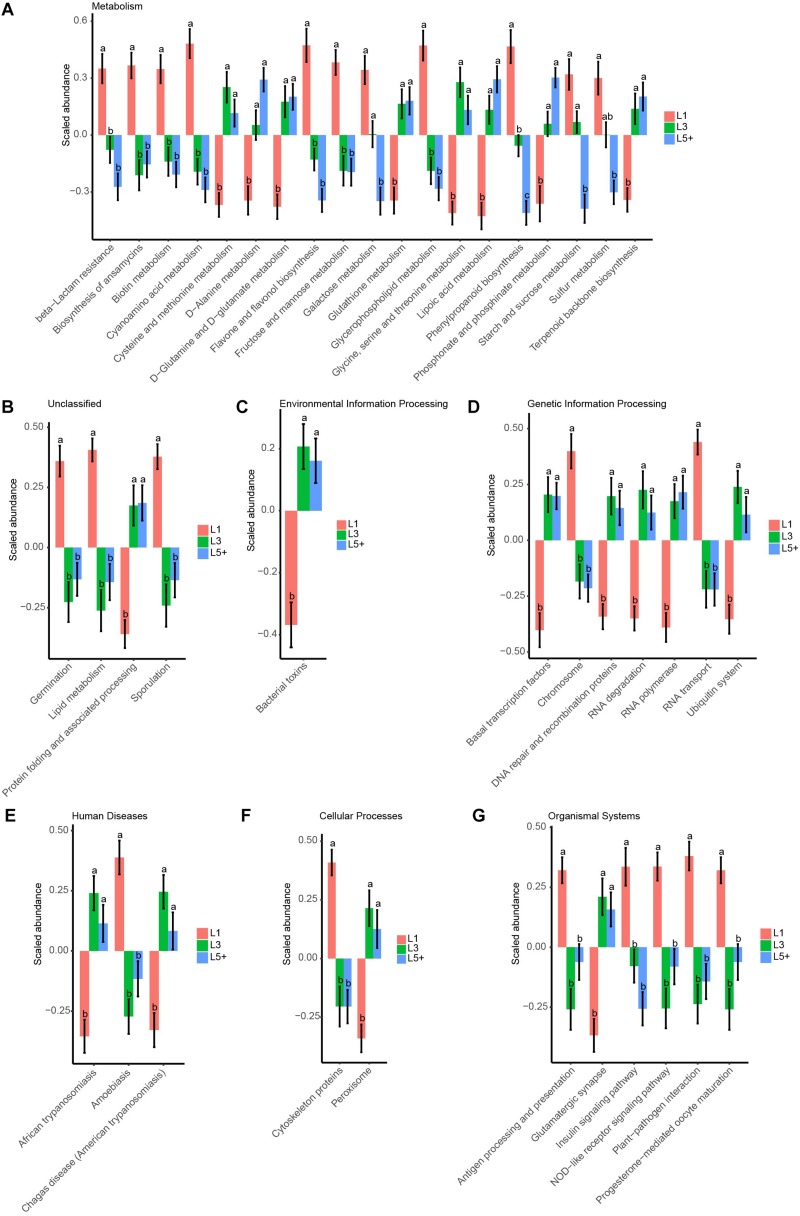
The 42 most significant KEGG pathways that were differently annotated across three lactation groups. ANOVA, followed by Duncan’s test, was used to assess the significance of the difference after log transformation and standard normal scaling of the relative abundance of the pathways. **(A–G)** The top 42 significant KEGG pathways with ANOVA *p*-values below 0.005 are showed in these barplots, which were split by the first taxonomic level of the KEGG pathways. The difference between two lactation groups with different letters above their bars was significant (Duncan’s test, *p* < 0.05), otherwise they were not significant, and the standard error (SE) of the group mean is displayed with error bars.

To study how the functions of fecal bacteria changed with increasing age, we first predicted the functions of fecal bacteria using PICRUSt, and then used ANOVA followed by Duncan’s test to compare the abundance of predicted KEGG pathways across the three lactation groups. We detected 249 KEGG pathways of the third level after filtering the pathways observed in less than 90 samples, which were not compatible with the analysis of ANOVA and Duncan’s test. We observed that 37% of the selected pathways (93 pathways) had changed during aging (ANOVA, *p* < 0.05). Figure 5 shows the 42 most significant pathways with *p*-values below 0.005. For the KEGG pathways related to essential nutrients shown in Figure 5, six pathways related to the metabolism of amino acids (Cysteine and methionine metabolism, D-Alanine metabolism, D-Glutamine, and D-glutamate metabolism, Glutathione metabolism, Glycine, serine and threonine metabolism, Phosphonate, and phosphinate metabolism) were more abundant in L5+ and L3 than in L1. Three pathways belonging to the metabolism of carbohydrates category (Starch and sucrose metabolism, Galactose metabolism, and Fructose and mannose metabolism) were more abundant in L1 than in L5+. Two pathways related to the metabolism of lipids (Glycerophospholipid metabolism and Lipid metabolism) were more abundant in L1 than in L3 and L5+. In addition, two pathways (NOD-like receptor signaling pathway, and Antigen processing and presentation) belonging to the Immune System category were less abundant in L3 and L5+. The biosynthesis of ansamycins appeared to be less active in L3 and L5+, and some pathways that might produce toxic metabolites such as, Peroxisome and Bacterial toxins, were more active in L3 and L5+. Two pathways related to trypanosomiasis (African trypanosomiasis and American trypanosomiasis) were more abundant in L3 and L5+ (Duncan’s test, *p* < 0.05).

### Correlation Analysis

**FIGURE 6:**
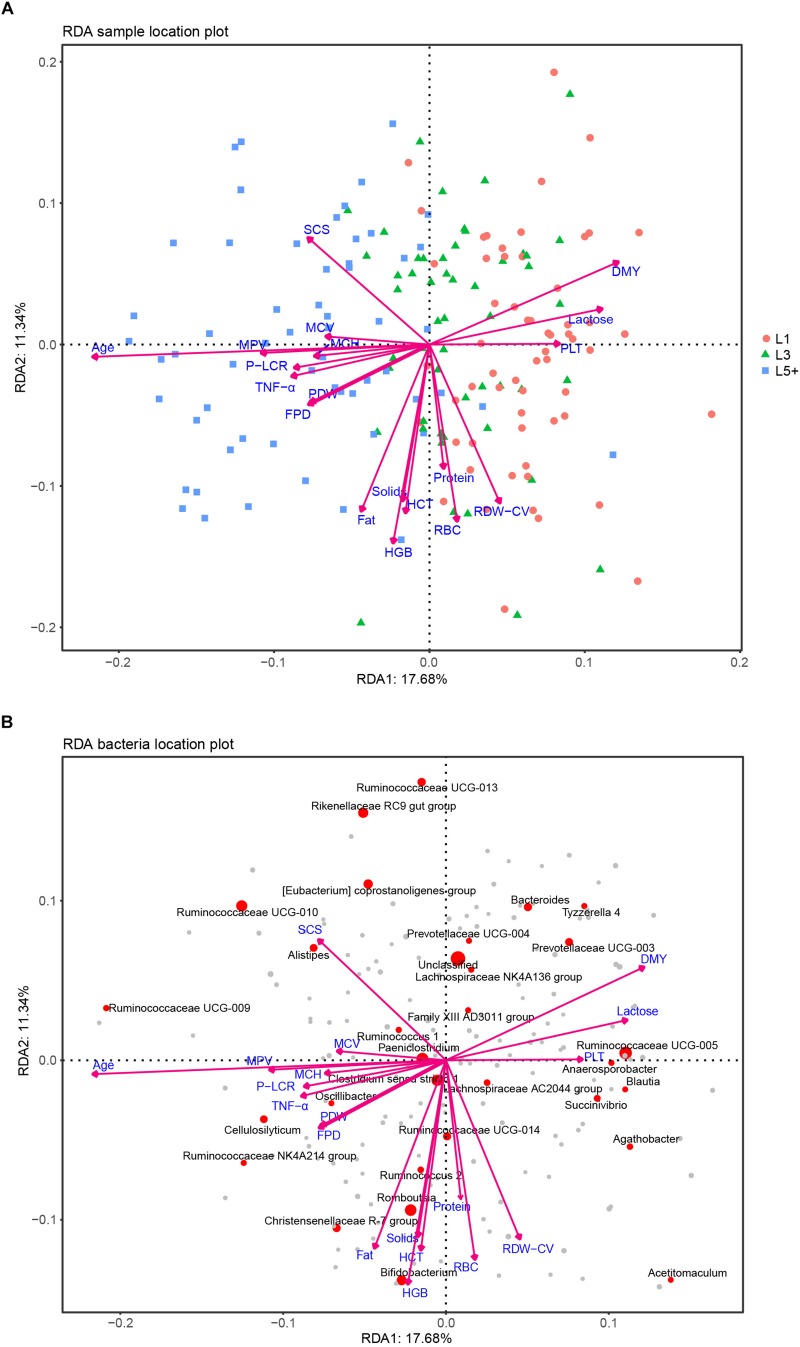
RDA analysis of the correlation between bacteria and a cows’ physiological status. **(A)** Each point represents a sample; each arrow represents a quantitative explanatory variable (age, inflammation-related cytokines, production performance, routine bloods). Projecting a sample’s point at right angles on an arrow approximates the position of the sample along that variable, and the distance between two samples’ points approximates their difference in bacterial communities; the cosine values of the angles between explanatory variables reflect their correlations. **(B)** Each point represents a bacterial genus; the species that failed to be assigned a genus taxa are included in “unclassified.” The top 30 abundant genera are labeled with their taxa. The size of a point reflects the abundance of the corresponding genus. The cosine values of angles between the bacteria and explanatory variables, and between the response variables (bacteria) themselves or explanatory variables themselves, reflect their correlations.

After the effects of farm groups were corrected, RDA was used to analyze how much the physiological indexes were related to the cows’ fecal bacteria community. The goodness of fit statistic of RDA is the squared correlation coefficient (*r*^2^), and the significance of this r^2^ was assessed using a permutation test in the VEGAN package. The results of performing 2000 permutations are shown in Table 4, which shows that age (months after birth), with a *r*^2^ of 0.353, was the variable most related to the bacterial community, except for farm groups, followed by DMY (0.143) and milk fat (Fat, 0.125). The variables with a significant r^2^ (*p* < 0.05) were used to draw RDA maps (Figure 6), in which the length of each arrow represents its degree of correlation to the bacterial community in the RDA (no permutations). Coloring the samples by their different lactation groups showed that the centroids of the three groups’ clouds were well separated, which confirmed the remarkable correlation between the cows’ age and their fecal bacteria community (Figure 6A). By plotting the bacteria in an RDA map, we could easily find the bacteria most related to a specific variable, and those most involved in the significance of the variable’s *r*^2^-value. For example, among the 30 most abundant bacteria labeled in Figure 6B, *Cellulosilyticum*, *Ruminococcaceae UCG-009*, and *Ruminococcaceae NK4A214 group*, located in the positive direction of the age arrow, were positively related to age, whereas *Succinivibrio*, *Ruminococcaceae UCG-005*, *Blautia*, *Anaerosporobacter*, *Agathobacter*, *Acetitomaculum*, and *Prevotellaceae UCG-003* were negatively related to age. In addition, these bacteria must contribute the most to the size of the *r*^2^-value for the age variable. More information could be found by comparing the angles between the arrows; for example, SCS, TNF-α, MPV, and MCH were positively related to age, as their arrows expanded toward almost the same direction; by contrast, platelets, daily milk yield, and milk lactose were negatively related to age.

**TABLE 4:** The correlation between physiological indexes and fecal bacteria communities.

**Index**	**r^2^**	***P-*value**
Age	0.353	<0.001
DMY	0.143	<0.001
Fat	0.125	<0.001
HGB	0.105	<0.001
Lactose	0.100	<0.001
Solids	0.098	<0.001
MPV	0.094	<0.001
HCT	0.076	0.002
RBC	0.073	0.002
SCS	0.070	0.003
P-LCR	0.064	0.003
RDW-CV	0.064	0.003
PLT	0.060	0.004
TNF-α	0.055	0.010
FPD	0.054	0.010
PDW	0.054	0.008
Protein	0.049	0.014
MCH	0.043	0.025
MCV	0.038	0.037
W-MCR	0.032	0.059
RDW-SD	0.031	0.077
IL-6	0.031	0.064
W-MCC	0.030	0.085
W-SCR	0.022	0.140
MCHC	0.022	0.150
W-LCR	0.022	0.139
TGF-β	0.019	0.207
IL-10	0.014	0.314
W-LCC	0.013	0.347
W-SCC	0.005	0.656
Car	0.004	0.746
WBC	0.002	0.816

*The significance (*p*-values) of the correlation between a cows’ physiological indexes and bacterial communities were assessed by permutating 2000 times using the method in the R VEGAN package. The goodness of fit statistic of RDA, the squared correlation coefficient (r^2^), which measures the correlation between cows’ physiological indexes and bacterial communities, is showed in this table. The abbreviations are the same as those shown in the footnote to Table 2.*

**FIGURE 7:**
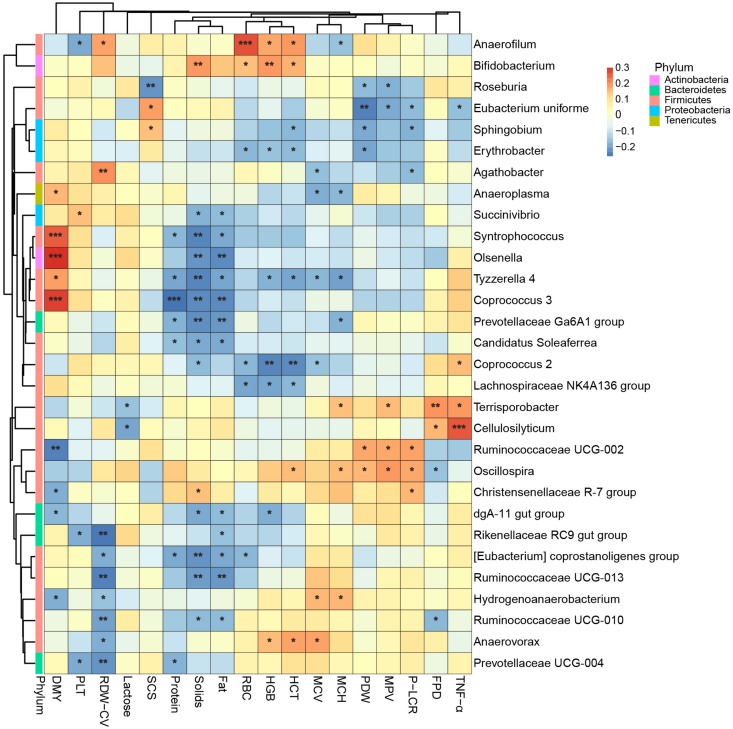
The 30 bacteria most related to cows’ physiological status. Spearman’s rank correlation is shown as the color of the tile in this heatmap: Red means a positive correlation, blue means a negative correlation. “^*^,” “^∗∗^,” “^∗∗∗^” indicate FDR (false discovery rate) adjusted *p*-values <0.05, <0.01, and <0.001, respectively.

Instead of relating bacteria to the physiological indexes while the effects of lactation groups existed, as above, we next removed the effects of both farm groups and lactation groups before we calculated the Spearman’s rank correlation between bacteria and physiological indexes. Therefore, the correlations shown in Figure 7 were more like partial correlations, meaning they were correlated even when the cows’ age was unchanged, and only the 30 bacteria most related to physiological indexes are shown. We found that *Cellulosilyticum*, which was more abundant in the L5+ group, was strongly and positively correlated to TNF-α. *Coprococcus 3*, which was more abundant in L3 compared with that in L1 and L5+, was strongly and positively correlated with daily milk yield [FDR (false discovery rate) adjusted *p* < 0.001].

### Further Association of the Ruminal Bacteria to the Lactation Group, and to Fecal Bacteria and Physiological Indexes

In this part, we were only able to analyze the samples of 30 cows from one of the six farms (F1) because of a lack of conditions to collect the ruminal liquid in the other farms. From the PCoA plot of the ruminal bacteria communities (Supplementary Figure S3), we observed the differences among the rumen bacteria communities of the three lactation groups (PERMANOVA, *p* < 0.05); however, compared with that of the L1 group, the rumen bacteria communities of L5+ were more similar to those of L3. In contrast to the LEfSe analysis of fecal microbiota, in this experiment we selected the bacteria with an LDA > 4 as the biomarkers of each lactation group in the LEfSe analysis of rumen bacteria to avoid false positives because we had only 30 samples. We found that, *Rikenellaceae RC9 gut group* and two genera belonging to the *Ruminococcaceae* (*Ruminococcaceae NK4A214 group* and *Ruminococcaceae UCG-014*) were more abundant in L3. The abundance of *Prevotella 1*, the most abundant bacterial genus in the rumen (average relative abundance = 0.34), declined when cows grew to the third lactation (L3). We did not find any ruminal bacteria biomarkers for the L5+ group in this study (Supplementary Figure S4).

**FIGURE 8:**
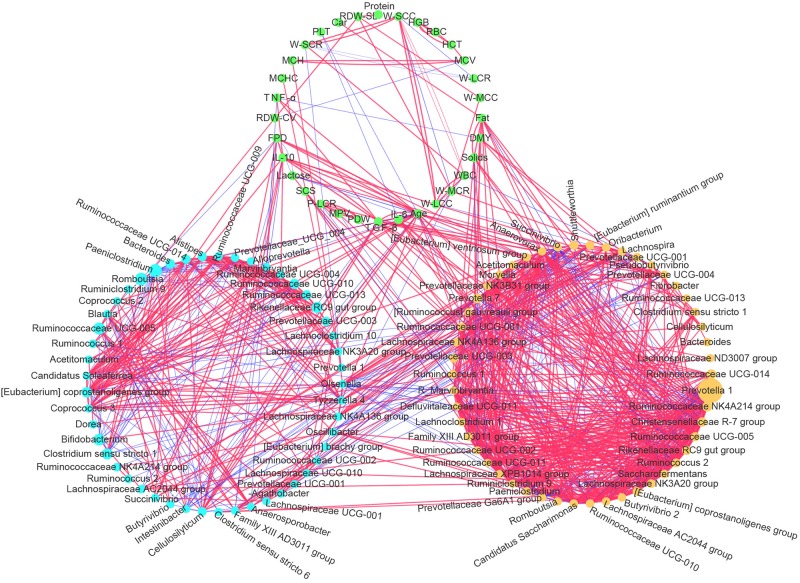
Correlation network among the most abundant 50 ruminal bacterial genera, most abundant 50 fecal bacterial genera, and physiological indexes (mean age, inflammatory cytokines, milk production performance, and routine bloods). Light blue nodes represent genera of fecal bacteria. Yellow nodes represent genera of ruminal bacteria. Green nodes represent the physiological indexes measured in this study. A line between two nodes represents their significant correlation (Spearman’s rank correlation, FDR adjusted *p* < 0.01), and the thicker the line is, the great the absolute value of the correlation coefficient. The red and blue lines represent positive and negative correlations, respectively. The sizes of the nodes of the ruminal and fecal bacteria reflect the abundance of the corresponding bacteria.

Correlation network analysis was used to visualize the Spearman’s rank correlation coefficient matrix among cows’ physiological indexes, the dominant rumen bacteria, and fecal bacteria. We detected a close and complex connection network among physiological indexes, rumen bacteria, and fecal bacteria (Figure 8). All the gut bacteria, whether in the rumen or feces, were connected with each other to some extent (directly or indirectly, positively or negatively). The interactions among rumen bacteria were much more complicated than those among fecal bacteria. Both rumen bacteria and fecal bacteria were related to the physiological indexes. Among the physiological indexes, the age variable had the largest number of connections with the ruminal and fecal bacteria (the number of lines connected to a node) in the network (32 connections in total), followed by TGF-β (17), IL-10 (13), and milk Fat (12), indicating that cows age and inflammatory cytokines play an important role in this network, and that gut bacteria were hypersensitive to increasing age and to the progress of inflammatory status. There were significant positive correlations among TGF-β, IL-10, TNF-α, and the cows’ age (FDR adjusted *p* < 0.01).

## Discussion

Chronic low-grade inflammatory status (inflammaging) was reported in humans and model animals (Claudio, 2010; Thevaranjan et al., 2017). In the present study, we observed similar inflammaging in older cows (L5+), with significantly higher levels of TGF-β, TNF-α, IL-10, and SCS (*p* < 0.001, Table 2). The L5+ group also showed decreased milk production performance, with significantly lower yields of milk and lower milk lactose levels compared with those in both L1 and L3 (*p* < 0.001, Table 2). Note that the increase in anti-inflammatory cytokines such as IL-10 should not always be interpreted as a beneficial change, because an increase of IL-10 might be a sign of dyshomeostasis as a result of increasing TNF-α (Figure 8). In addition, anti-inflammatory cytokines can have proinflammatory activity under specific circumstances. Inflammaging is thought to be the cause of the age-related decline in the functionality of the immune system (immunosenescence) (Larbi et al., 2008). Thus, it is not difficult to explain the negative effect of TNF-α on milk yield in the current study (Figure 1). Inflammaging may be one of the reasons for higher culling hazard and reduced production efficiency in older cows.

Age-related changes in the composition of the symbiotic microbiota in humans have been demonstrated (Biagi et al., 2010; Liang et al., 2011; Yu et al., 2015); however, the results of these studies differed in terms of signature microbes and inconsistent conclusions. In fact, mice under different experimental conditions also showed different changing pattern with increasing age (Kozik et al., 2017; Thevaranjan et al., 2017). Moreover, Zaneveld believed that the microbiological changes induced by many perturbations are stochastic, and thus lead to a transition from a stable to an unstable community state (known as the “Anna Karenina principle” for animal microbiomes) (Zaneveld et al., 2017). Thus it is important to conduct experiments under different conditions to form a valid conclusion. In the current study, we observed marked differences among the fecal microbiota derived from cows reared in different farms (Supplementary Figure S1). This suggested that the effect of farms, which might be co-effects of the management action, the composition of the total mixed ration, and environment of the different farms, was one of the reasons that led to the changes in pattern of the cows’ feces microbiota.

In the analysis of large data derived from various environments, there is a lack of methods to handle an experiment design comprising more than one category variable as an influencing factor on microbes whose abundance was known to disobey the Gaussian distribution. This rules out any analysis model that considers Gaussian distribution as a fundamental assumption. In the present study, we used a non-parametric method (Johnson et al., 2007; Leek et al., 2012) to remove the effect of the uninterested variable (farm), and used another non-parametric method, LEfSe (Segata et al., 2011), to test the effect of the variable of interest (lactation group), thus producing believable results and conclusions.

By conducting an experiment upon 180 cows from six different farms, we were able to draw robust conclusions for the different farm environments. We found that the bacterial community did indeed change with increasing age (Figure 2 and Table 3), and some bacteria that were present extensively in the six farms displayed a relative stable changing pattern across the lactation groups (Figures 3, 4). In particular, the bacteria belonging to the *Prevotellaceae* were more abundant in both the rumen and feces of young dairy cows (L1) compared with those in L3 and L5+ (Figure 3 and Supplementary Figure S4), which was consistent with the study of Liu (Liu et al., 2017). Dysbiosis of fecal microbiota could definitely be related to inflammation (Figure 8); for example, *Cellulosilyticum*, which was strongly and positively related to TNF-α and negatively related to milk lactose when the effect of lactation group was removed (Figure 7), was more abundant in L5+ (Figure 3), indicating that it might be the key reason for the inflammation of the older cows (L5+).

Our study also demonstrated dysbiosis of the fecal bacteria community in the older cows by comparing the predicted functions across the three lactation groups. The reconfiguration of microbiota in older cows led to changes in the metagenome such that it contained more functions related to protein metabolism and fewer functions related to carbohydrate and lipid metabolism (Figure 5). This finding was in accord with a previous study of the human metagenome (Rampelli et al., 2013). The fermentation of proteins often leads to the production of toxic chemical substances such as NH_3_, H_2_S, amines, and phenols (Brüssow, 2013), while the loss of lipid and carbohydrate related genes may decrease the potential to generate beneficial compounds, such as short chain fatty acids (SCFA), which can protect the intestinal tract from damage. Some species belonging to the *Lachnospiraceae*, for example, were reported to protect against colon cancer in humans by producing butyric acid (Meehan and Beiko, 2014). In the present study, we detected a decrease of many genera belonging to *Lachnospiraceae* (14 genera) among cows in the L5+ group compared with those in either the L1 or L3 groups, in both the feces and rumen (Figure 3 and Supplementary Figure S4). Although not all the species belonging to *Lachnospiraceae* showed a beneficial effect on host health in our study (e.g., *Cellulosilyticum*), *Coprococcus 3*, in particular, was more abundant in cow feces of the third lactation (Figure 3), as well as strongly and positively related to milk yield (Figure 7), indicating that *Coprococcus 3* might be related to the high milk production of third lactation cows. Moreover, Brüssow (Brüssow, 2013) classified the *Bacteroidaceae*, *Eubacterium*, and *Bifidobacterium* as beneficial bacteria, because of their ability to synthesize vitamins, help in digestion, stimulate immune function, and inhibit pathogenic microbes. However, these bacteria showed a lower abundance in cows in the L5+ group compared with those in either the L1 or L3 group (Figures 3, 4). Certain predicted KEGG pathways, which tend to have negative effect to host health, such as Peroxisome, Bacterial toxins, African trypanosomiasis, and American trypanosomiasis, showed a higher abundance among dairy cows in the L3 and L5+ groups than in the L1 group (Figure 5). The increase of the peroxisome pathway in older cows may lead to the production of H_2_O_2_. In addition, *Hydrogenoanaerobacterium* is an H(2)-producing anaerobic bacterium, and the increase in its abundance in cows in the L5+ group (Figure 3) might lead energy waste and the production of H^⋅^ free radicals. As reactive oxygen species (ROS), H_2_O_2_ and H^⋅^ could cause oxidative stress (OS), leading to further inflammation. Therefore, on the one hand, older dairy cows, especially cows of L5+, face more threats from toxic substances and ROS, both of which can cause morbidity of older cows. On the other hand, older dairy cows lack of the protection of short chain fatty acids, which increases the chances of toxic compounds and ROS damaging the intestinal mucosa and further entering blood circulation. In summary, a decayed microbiota might be one of the reasons why older dairy cows suffer from chronic low-grade inflammation and decreased milk production.

Our study mainly focused on the bacterial community of feces (180 samples), but we also related fecal bacteria to rumen bacteria (30 samples). Rumen bacteria reacted to aging similarly to fecal bacteria: *Prevotellaceae* and *Lachnospiraceae* showed a similar changing pattern between rumen bacteria and feces bacteria. However, rumen bacteria showed some differences; for example, the interactions among rumen bacteria were much more complicated than those among fecal bacteria (Figure 8). All the gut bacteria (in both the rumen and feces) were related to each other (directly or indirectly, positively or negatively) (Figure 8), which made them a community, and it is possible that any changes in its members would cause a domino effect. Our study supports the hypothesis that the fecal microbiota plays an important role in host health, at least as an indicator of host health, suggesting that we should pay more attention to the balance of the rectal microbiota.

Given the existence of methods to manipulate the gut microbiota, such as fecal microbiota transplantation (FMT) (Weingarden et al., 2015), dietary intervention (Weimer, 2014), or feeding with probiotics directly, the question remains as to which microbes should be our focus. The results of the present study support the hypothesis of prolonging a cows’ productive life and improve dairy cow milk productive performances by manipulating the gut microbiota. Manipulating the levels of species belonging to the *Lachnospiraceae*, *Bacteroidaceae*, *Eubacterium*, *Bifidobacterium*, *Hydrogenoanaerobacterium*, which showed a close connection to the cows’ age and either milk production or inflammatory cytokines, might help to alleviate inflammaging and boost milk production. Some bacteria, such as the *Prevotellaceae*, which showed a stable changing pattern with aging in various circumstances, should be subjected to further intensive study to confirm their influence on host health.

## Data Availability

The datasets generated for this study can be found in NCBI Sequence Read Archive, SRP202074.

## Ethics Statement

The Institutional Animal Care and Use Committees (IACUCs) approved all the experimental procedures, which complied with the China Physiological Society’s guiding principles for research involving animals.

## Author Contributions

YCW, YJW, and GZ conceived and designed the experiments. GZ, HL, WQ, LH, HZ, GD, GG, and YJW contributed to the samples collection and conducted the experiments. GZ analyzed the measured data and wrote the first draft. YCW critically reviewed the manuscript.

## Conflict of Interest Statement

GZ was employed by the Shenzhen Weishengtai Technology Co., Ltd. (Shenzhen, China). GD and GG were employed by the Beijing Sunlon Livestock Development Co., Ltd. (Beijing, China). The remaining authors declare that the research was conducted in the absence of any commercial or financial relationships that could be construed as a potential conflict of interest.

## Supplementary Material

FIGURE S1 Differences among the dairy cows’ feces microbiota at six farms. **(A)** The unsupervised PCoA of Bray–Curtis dissimilarity. Each point represents a sample with the composition structure of all observed sOTUs; the distance between two points thus represents the difference in the microbiota between two corresponding samples. **(B)** Flower figure of the distribution of sOTUs among the six farms. The number in each petal represents the number of sOTUs that are peculiar to the corresponding farm, and the number in the center represents the number of sOTUs that are common for all six farms.

FIGURE S2 Histogram of the LDA scores computed for the differentially abundant fecal bacteria among the six farms. The bacteria biomarkers of different farms are represented with different colors.

FIGURE S3 Principal coordinates analysis (PCoA) to visualize the differences among ruminal bacteria communities of three lactation groups within the first farm (F1). Each point represents a sample in F1; the distance between two points approximates the difference of their bacterial communities (Bray–Curtis dissimilarity), and the points belonging to different lactation groups were shown in different colors.

FIGURE S4 LEfSe (Linear discriminant analysis effect size) to identify the ruminal bacteria biomarkers of three lactation groups. **(A)** Histogram of the LDA scores computed for differentially abundant rumen bacteria across three lactation groups. **(B)** The ruminal bacteria (highlighted by small circles and by shading) showing different abundance values among the three lactation groups.

TABLE S1 SOTUs specifically present in a farm or commonly distributed among all six farms. There are seven sheets in this Excel file; the first sheet exhibits the sOTUs commonly distributed among all six farms; the second to seventh sheets show the sOTUs specifically present in each farm; each sheet shows the total sequence count and taxon of each sOTU.
