# Supplementary figures and images for: The Association Between Inflammaging and Age-Related Changes in the Ruminal and Fecal Microbiota Among Lactating Holstein Cows
Source: Front Microbiol. 2019 Aug 9;10:1803. doi: 10.3389/fmicb.2019.01803 (PMC6696898; doi:10.3389/fmicb.2019.01803)

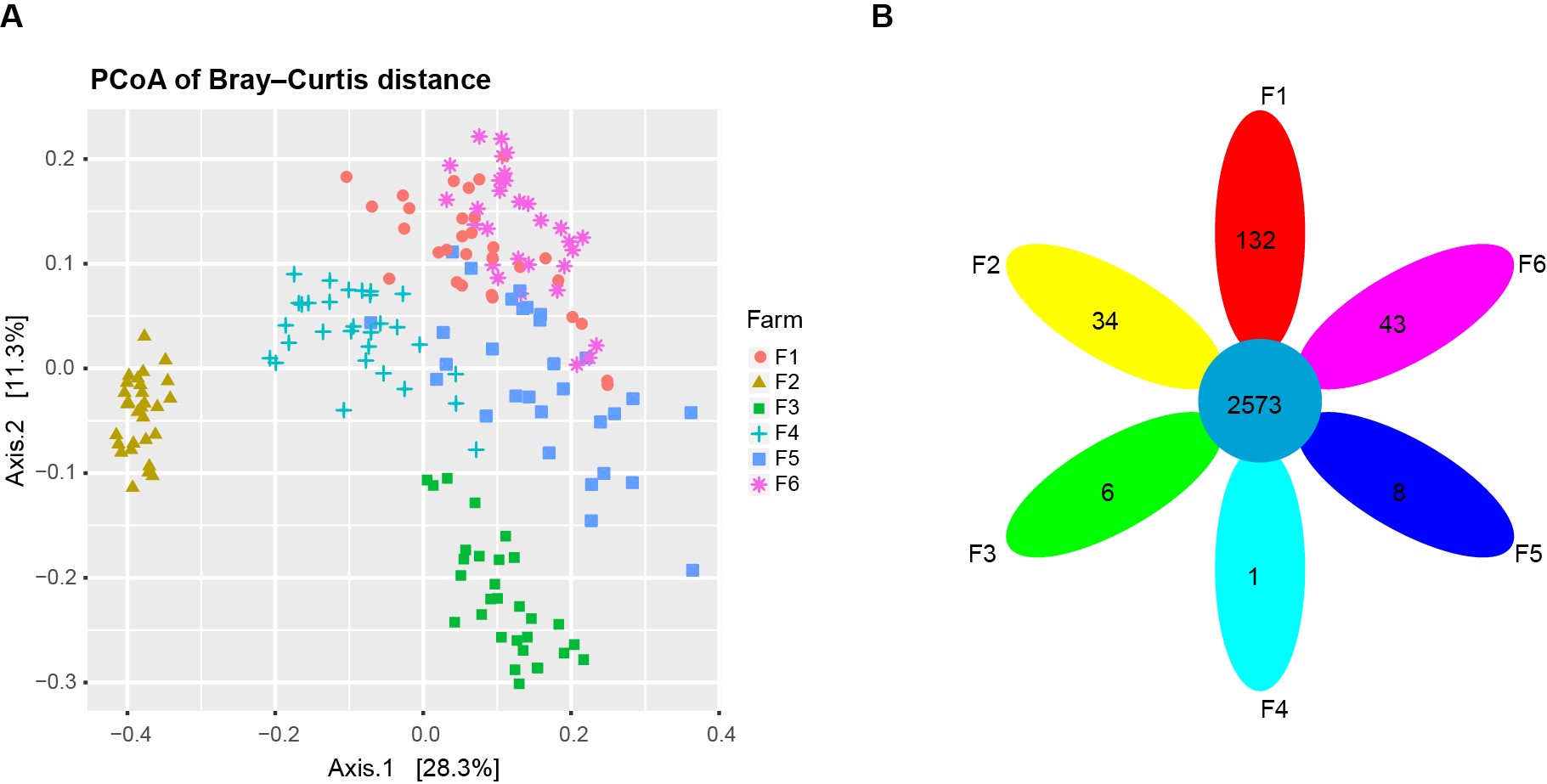

Supplement: FIGURE S1 — Differences among the dairy cows’ feces microbiota at six farms. (A) The unsupervised PCoA of Bray–Curtis dissimilarity. Each point represents a sample with the composition structure of all observed sOTUs; the distance between two points thus represents the difference in the microbiota between two corresponding samples. (B) Flower figure of the distribution of sOTUs among the six farms. The number in each petal represents the number of sOTUs that are peculiar to the corresponding farm, and the number in the center represents the number of sOTUs that are common for all six farms. [file Image_1.JPEG]

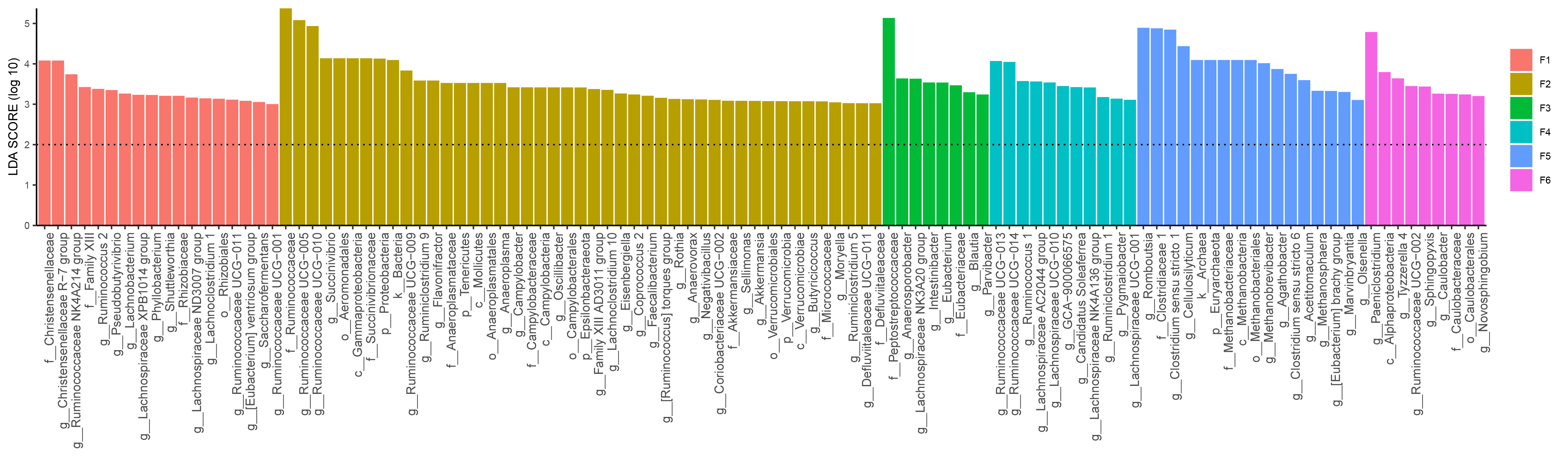

Supplement: FIGURE S2 — Histogram of the LDA scores computed for the differentially abundant fecal bacteria among the six farms. The bacteria biomarkers of different farms are represented with different colors. [file Image_2.JPEG]

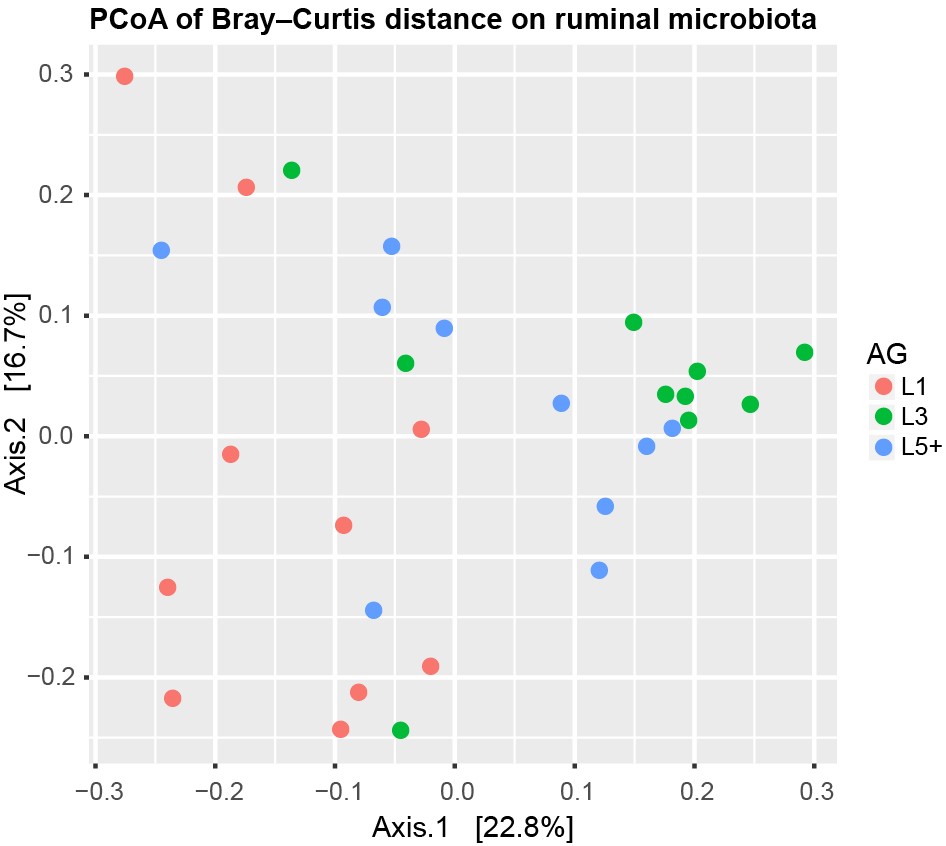

Supplement: FIGURE S3 — Principal coordinates analysis (PCoA) to visualize the differences among ruminal bacteria communities of three lactation groups within the first farm (F1). Each point represents a sample in F1; the distance between two points approximates the difference of their bacterial communities (Bray–Curtis dissimilarity), and the points belonging to different lactation groups were shown in different colors. [file Image_3.JPEG]

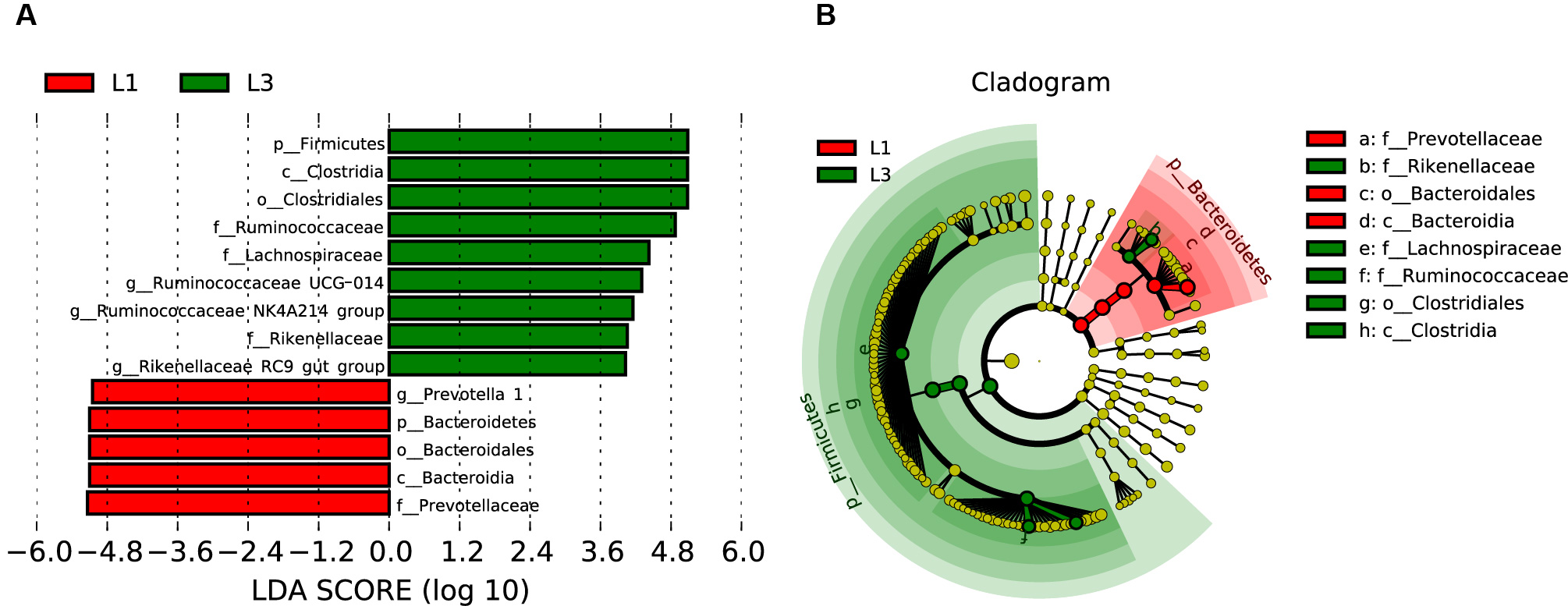

Supplement: FIGURE S4 — LEfSe (Linear discriminant analysis effect size) to identify the ruminal bacteria biomarkers of three lactation groups. (A) Histogram of the LDA scores computed for differentially abundant rumen bacteria across three lactation groups. (B) The ruminal bacteria (highlighted by small circles and by shading) showing different abundance values among the three lactation groups. [file Image_4.JPEG]
